# Cancer Salt Nostalgia

**DOI:** 10.3390/cells10061285

**Published:** 2021-05-21

**Authors:** Aashish S. Allu, Venkataswarup Tiriveedhi

**Affiliations:** 1 Department of Sciences, Lafayette High School, Wildwood, MO 63011, USA; aallu021@rsdmo.org; 2 Department of Biological Sciences, Tennessee State University, 3500 John A Merritt Blvd, Nashville, TN 37209, USA; 3 Division of Pharmacology, Vanderbilt University, Nashville, TN 37232, USA

**Keywords:** cancer biology, salt, immunotherapy, T-helper cells, cytokines, sodium channels

## Abstract

High-salt (sodium chloride) diets have been strongly associated with disease states and poor health outcomes. Traditionally, the impact of salt intake is primarily studied in cardiovascular diseases, hypertension and renal diseases; however, recently there has been increasing evidence demonstrating the role of salt in autoimmune diseases. Salt has been shown to modulate the inflammatory activation of immune cells leading to chronic inflammation-related ailments. To date, there is minimal evidence showing a direct correlation of salt with cancer incidence and/or cancer-related adverse clinical outcomes. In this review article, we will discuss the recent understanding of the molecular role of salt, and elucidate the apparent double-edged sword nature of the relationship between salt and cancer progression.

## 1. Introduction

Solid tumors are known to have a higher sodium concentration when compared to the surrounding soft tissue [1]. Sodium magnetic resonance imaging (^23^Na-MRI) scans can provide a qualitative and semi-quantitative measure of tissue sodium concentration [2]. In acute strokes, the extent of brain injury and tissue viability is clinically assessed utilizing the tissue sodium concentration measurements using ^23^Na-MRI [3]. In murine models of glioma and prostate cancer, ^23^Na-MRI has shown tumor tissue to have a higher concentration of sodium along with a probable chemotherapy-induced modulation of tumor sodium concentration [4,5]. ^23^Na-MRI studies in human breast cancer patients have shown a tumor sodium content of 30–70% above the surrounding soft tissue [6,7,8].

In human tissue, the sodium ion [Na^+^] concentration is approximately ten times higher in the extracellular compartment compared to the intracellular fluid. While the sodium concentration in extracellular fluid [ECF-Na^+^] ranges from 135 to 145 mM, the intracellular fluid sodium concentration [ICF-Na^+^] is 5–15 mM. This difference is actively maintained by an ATP-dependent Na^+^/K^+^ pump [9]. The free movement of sodium between the ICF and ECF compartments is restricted by the cell membrane. Overall tumor sodium concentration is a weighted average of the intracellular and extracellular sodium concentrations. Among the total number of ion transporters and channels, up to 90–95% are potassium channels and only 1–2% are sodium channels. Similar to sodium channels, chloride and calcium channels also account for up to 2% each of the entire ion channels on the cell membrane [10,11]. The intracellular influx of sodium is associated with an osmotic movement of water that contributes to the cell swelling usually noted during various cellular death processes [12,13]. Due to its apparent inflammatory role, there is a well-established correlation between salt and several disease states, including hypertension, stroke, cardiac and renal diseases [14,15,16]. However, there is no direct correlation between salt and cancer. In this review article, we will discuss the recent molecular and mechanistic understandings of the role of salt in tumor microenvironments.

## 2. Salt Induced Tumorigenesis

Chronic inflammation is one of the established hallmarks of cancer development [17]. A chronically inflamed microenvironment can be induced either by reactive oxygen/nitrogen species (ROS/RNS) [18], paracrine factors, or tumor-infiltrating cells, inciting continuous cell proliferation, DNA damage, or cancer transformation [19]. Inflammatory cytokines [20,21,22] and chemokines [23,24,25] provide conducive signaling to induce cancer proliferation [20,26] and tumor angiogenesis [17]. It is well-established that salt induces a chronic inflammatory response [27,28]. Cancers are known to have multifactorial etiology [29,30]. The relationship between hypertension and cancer has been a considerable area of debate for the past five decades. A seminal landmark prospective study by Dyer et al. in 1975 provided evidence for a potential causal association between cancer and hypertension [31,32]. Several population studies over the subsequent five decades provided conflicting evidence, with some studies arguing in favor of [32,33], and others arguing against, an association between hypertension and cancer [34], but no consensus was reached. Although a direct correlation between a high-salt diet and breast cancer is not readily evident from the scientific literature, a strong correlation between hypertension and salt-sensitivity is well-accepted. Abnormal upregulation of cell proliferative pathways along with reduced apoptosis has been noted in hypertension [35]. Further, it is unclear if the enhanced innate salt sensitivity observed in some demographics [36] will play a role in tumor sodium accumulation or salt-mediated carcinogenesis.

In 1971, Dr. Clarence D. Cone Jr. proposed that sustained depolarization of the cell membrane may induce mitogenic activity [37]. At that time this theory was vigorously debated, and importantly this theory had limited experimental evidence supporting the mechanistic basis for ionic changes leading to sustained depolarization. Interestingly, after a decade, in 1981, Nagy et al. used energy-dispersive X-ray microanalysis on human tumor tissue biopsy specimens, revealing a three-fold increased intracellular sodium content along with a five-fold increase in Na^+^-to-K^+^ ratio in cancer cells as compared to normal cells [38]. Around the same time, in 1983, Sparks et al. demonstrated that hepatomas and mammary adenocarcinomas had higher intracellular sodium concentration as compared to a normal liver and lactating breast epithelium, respectively [39]. Further, the same research group showed amiloride, a potassium-sparing epithelial sodium channel inhibitor used as a diuretic in anti-hypertension therapy [40], reduced tumor growth and cellular proliferation [41,42]. From these original studies it is unclear whether the cause of tumor activity is directly due to high intratumor salt concentration or if it is a mere association.

Voltage-gated sodium channels (VGNaC) allow the movement of sodium ions along the concentration gradient through the cell membrane. Overexpression of VGNaC is associated with cell proliferation and metastasis in colorectal cancer [43], oral squamous cell carcinoma [44], non-small cell lung cancer [45], breast cancer [46], and cervical carcinoma [47]. The expression of a SCN9A, another VGNaC, in prostate cancer is associated with a higher risk of metastasis and is used as a clinical biomarker [48]. Aquaporins (AQP) are water channels that regulate serum sodium concentration with aggressive behavior in breast cancer [49], liver cancer [50], esophageal cancer [51] and gastric cancer [52]. It is also important to note that the expression and membrane localization of ion exchanger channels, such as the sodium/calcium exchanger (NCX) that enables the bi-directional movement of calcium and sodium ions based on net electrochemical gradient, were increased in leukemia, esophageal cancer, pancreatic cancer and breast cancer [53,54].

Studies from our laboratory have shown that the use of high-salt treatments on breast cancer cells induced upregulation of the expression of inflammatory epithelial sodium channels (γENaC), leading to ERK-1/2 transcription factor-dependent cell proliferation and the production of reactive nitrogen and oxygen species (RNS/ROS) [55]. Studies from other research groups have also demonstrated the ENaC-mediated proliferation and migration of hepatoma cells [56]. Further, amiloride-sensitive ENaC channels are known to induce oncogenesis in high-grade gliomas such as glioblastoma multiforme [57,58]. All these data suggest the direct functional role of ENaC in high-sodium-induced cancerous development. High salt levels have also been shown to enhance the expression of P-glycoprotein channels, which pump chemotherapeutic agents out of the cell, resulting in chemoresistance in cancer [59].

Normal cells perform glucose metabolization using mitochondrial oxidation to yield 36–38 ATP. However, due to higher anabolic needs, a cancerous cell under similar normoxic conditions performs glycolysis, which yields only 2 ATP, but it is also important to produce the metabolic by-products needed to produce cellular building blocks, a process referred to as aerobic glycolysis or the Warburg effect [60,61]. A high sodium concentration is shown to enhance the Warburg-like metabolism in cancer cells as evidenced by enhanced glucose consumption in breast cancer cells [62]. Tumor neo-angiogenesis is crucial for cancer metastasis. The vascular endothelial growth factor (VEGF), through induction of AkT/PI3k signaling, plays an important role in angiogenesis. Our studies have demonstrated that high salt levels enhanced the expression of VEGF in breast cancer cells through the nuclear factors of activated T cells (NFAT5) signaling [63].

**Figure 1:**
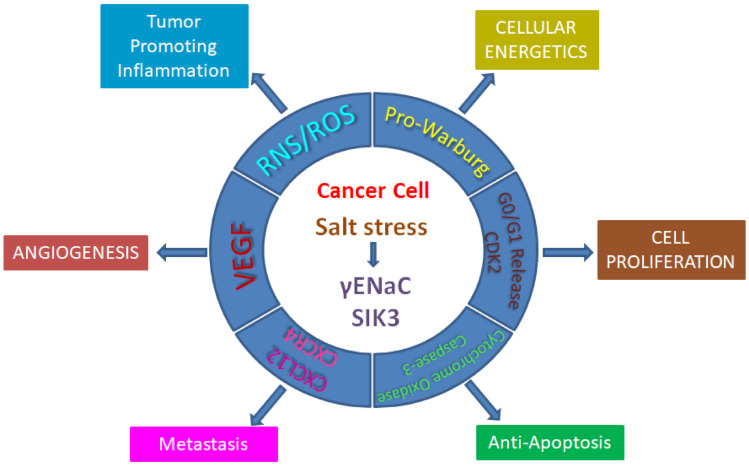
Impact of high salt on hallmarks of cancer.

Our phosphoproteomic analysis identified a unique molecular target, salt-inducible kinase-3 (SIK-3), that is specifically upregulated in breast cancer cells following high-salt treatment [64]. Upon high-salt treatment, the mTORC2 protein complex works upstream to phosphorylate serine-493 of SIK3 and activates it. Specifically, high-salt treatment played a critical role in the SIK3-mediated G0/G1-phase release of the cell cycle, leading to enhanced mitogenicity. Further, SIK3 played a critical role in arginine metabolization, wherein high-salt treatment induced the enzymatic activation of proinflammatory inducible nitric oxide synthetase (iNOS) and arginosuccinate synthetase (ASS-1), leading to the production of reactive nitrogen species. High-salt-induced SIK3 also played a critical role in enhancing the surface expression of CXCR4 chemokine receptors, which is important for tumor metastasis. Taken together, all these data provide molecular evidence to strongly suggest that high salt in the tumor microenvironment induces cancer cell proliferation and metastasis (Figure 1).

## 3. Salt: A Double-Edged Sword in Tumorigenesis

Multiple lines of evidence from pre-clinical and human studies have established the dual and apparently antagonistic role of the immune system, popularly called immunoediting [65]. During the initial phase, the immune system detects nascent transformed or dysplastic malignant cells and eliminates them by a process referred to as immunosurveillance. At later phases, the immune system performs a complete 180-degree turn and promotes tumor progression and the development of tumor variants through a process referred to as immune escape [66,67]. As the tumor progresses to an increasingly aggressive phenotype, immunosuppressive mechanisms predominate, and unfortunately most of the immune checkpoint inhibitor therapies specifically designed to activate host’s adaptive immunity have already been blunted, possibly limiting their clinical success [68]. In this review, we will consider the role of salt in these various phases of immune-editing.

While a high-salt diet due to tonicity stress and mitogenicity (as discussed earlier) would be expected to enhance tumor progression, slightly counterintuitively to this logic, a high-salt diet in preclinical models inhibited tumor progression. Studies by Willebrand and colleagues [69], along with evidence from He and colleagues [70], have demonstrated that a high-salt diet in murine breast and melanoma tumor models has resulted in diminished tumor progression along with the inhibition of immunosuppressive myeloid-derived suppressor cells. We have also seen similar results in the pre-clinical immunocompetent murine breast tumor models. High salt is known to induce pro-inflammatory immune responses. The treatment of naïve CD4 + T lymphocytes with an additional 40 mM NaCl to in vitro culture medium induced an inflammatory Th17 phenotype switch through p38/MAPkinase transcription factor activation, mediated by SGK1 and NFAT5 signaling mechanisms [71,72,73]. Further, high salt has been shown to induce inflammasome upregulation resulting in caspase-1 activation, leading to the cleavage of pro-IL1β to active IL1β. Further, high salt has also been shown to cause the polarization of anti-inflammatory CD4 + FoxP3 + regulatory T cels (Treg) to the inflammatory Th1 phenotype with the secretion of inflammatory cytokine IFNγ [74,75,76]. Further, a high-salt diet has been shown to exacerbate autoimmune disease states such as experimental autoimmune encephalitis (EAE) and graft versus host disease (GvHD) in pre-clinical models [71,76]. This leads us to think that a high-salt diet in preclinical cancer models activates the immune elimination phase of the immune editing process.

**Figure 2:**
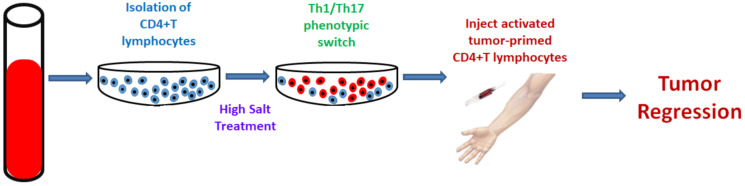
Anti-tumor effect of ex vivo high-salt-activated tumor-primed CD4 + T cells.

A general high-salt dietary recommendation to promote anti-tumor immune responses would not be practical. We have recently shown that ex vivo high-salt treatment of tumor-primed CD4 + T cells resulted in effector (Th1 and Th17) phenotype activation, and also exerted a strong anti-tumor effect (Figure 2). The CD4 + T lymphocytes collected from tumor-draining lymph nodes were ex vivo activated with high-salt treatment [77]. We have shown that this high-salt treatment induced the initial effector phenotypic switch of CD4 + T cells, which was followed by a delayed immune exhaustion. The effector phenotype switch is associated with higher glycolytic oxidation rates, suggesting that the incomplete oxidation of glucose produces bi-products needed for the anabolic processes required for cell growth, the production of pro-inflammatory proteins such as cytokines, and lipid synthesis for cell membrane and endomembrane organelle support. On the other hand, the anti-inflammatory regulatory CD4 + T cells (Treg) have been shown to have a higher mitochondrial oxidation rate. However, continued treatment with high salt levels resulted in immune exhaustion and immunosuppressive phenotypic changes in ex vivo high-salt-treated CD4 + T cells. 

**Figure 3:**
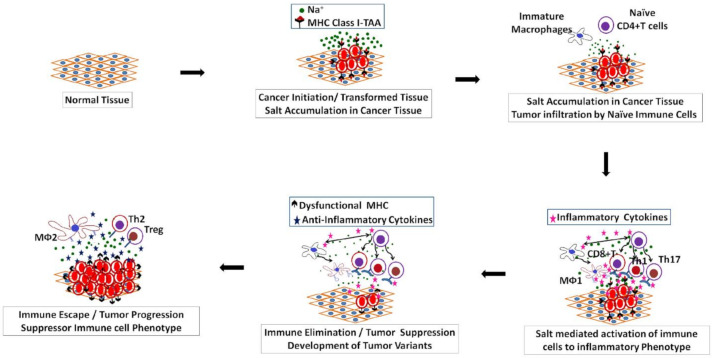
Schematic to demonstrate the overall impact of salt on tumor immune editing. Specifically, we postulate that salt accumulation in tumor tissue is the first protective host response against cancerous/dysplastic changes. High salt in the tumor microenvironment leads to the inflammatory activation of tumor-infiltrating naïve CD4 + T cells to anti-tumor cytotoxic Th17/Th1-lymphocytes, and also the differentiation of tumor-infiltrating macrophages to the inflammatory MΦ1-macrophage phenotype leading to the tumor immune elimination phase. In later phases, there is tumor progression with the development of immune exhaustion and immune-resistant tumor variants.

While in the initial phase of tumor growth salt promotes tumor elimination by immune surveillance, the impact of salt in the delayed phase of tumor growth is still unclear. As mentioned above, our in vitro studies with CD4 + T cells have demonstrated that continued high-salt treatment resulted in CD4 + T cell exhaustion [77]. Similarly, the prolonged in vitro treatment of human monocytes with high salt resulted in an M2 macrophage phenotypic switch, which is anti-inflammatory and pro-tumor [78]. These data suggest that in the latter phases of tumor immunoediting, salt could perform a pro-tumor immunogenic role by promoting immune exhaustion (Figure 3). However, developing a precise pre-clinical model to verify this hypothesis is challenging. Most of the syngeneic orthotopic tumor implant models involve the injection of cancers in the flank or subcutaneous tissue and analyzing tumor progression following a high-salt diet. In the initial phase, due to the salt-induced inflammatory activation of immune cells, there is heightened immune surveillance leading to tumor elimination and reduced tumor progression. This temporal study usually lasts 4–8 weeks in preclinical murine models. However, continuing these experiments to later phases of immune escape will be a challenge, as the salt would have induced secondary generalized inflammatory changes in the murine model, and under ethical humane practices murine experiments cannot be continued for the 3–6 months required to determine the immune escape role of salt. Therefore, immunocompetent syngeneic murine models might not serve as good experimental models to study the delayed tumor response to a high-salt diet. In the real-world human setting, a high-salt diet is usually a chronic phenomenon, and tumor growth in patients might be a result of a tumor overcoming the initial tumor elimination phase and later phase immunosuppression. More precise future studies with a more appropriate preclinical model should be developed to specifically study the delayed impact of salt in the tumor microenvironment. Further, as immune checkpoint inhibitors work by activating immune cells, the combinatorial role of a high-salt diet and immune checkpoint inhibitors on tumors needs to be evaluated.

## 4. Application of Sodium MRI as a Prognostic and/or Diagnostic Marker

The measurement of tumor sodium concentration has two components: the extracellular and intracellular sodium concentrations. The total sodium concentration in the tumor would be a weighted average of these two components. In preclinical models, total tumor sodium is analyzed by dry-ashing or nitric acid dissolving followed by spectrometry measurements, such as atomic absorption or X-ray fluorescence. Alternatively, more quick and easy techniques, such as ion electrode measurement, could be used. All these methods report data from wet tissue or dry tissue measurement with varying levels of repeatability and reproducibility. However, all these methods require a significantly large tissue sample, of either approximately 0.5 cm in diameter or a tumor tissue volume of around 50–70 μL [79,80,81,82,83]. Further, these techniques cannot differentiate between extracellular versus intracellular sodium concentrations. Sodium MRI (^23^Na-MRI) will be a reasonable non-invasive procedure that can be adopted for human cancer healthcare [84]. ^23^Na-MRI is already a clinically approved procedure to determine brain tissue viability following an acute stroke. Importantly, ^23^Na-MRI uses an inversion recovery pulse sequence that utilizes the difference in T_1_ relaxation times of free and bound sodium. While ^23^Na-MRI could be difficult to apply to abdominal tumors due to radiofrequency decay and the proximity of the kidney, which inherently has a high sodium concentration, on other solid organs (such as breast, lung, prostrate etc.), this is a safe, quantitatively reliable technique with a low scan-time when used on other solid tumors in clinical settings [5,85]. Recently, for brain imaging, a 3D-cones trajectory approach was adopted to improve acquisition times compared to the more commonly utilized 3D-radial *k*-space sampling method [86,87]. Although not well-established, it can be envisioned that sodium concentration in the extracellular tumor space could exert an osmotic stress effect on cancer cells, with intracellular sodium being responsible for the inflammatory responses in cancer and immune cells in the tumor microenvironment. The ability of ^23^Na-MRI to differentiate between intra- and extracellular could be probably used to determine the benign versus aggressive solid tumors and also probably to determine the chemosensitivity or chemoresistance of the tumor. More future clinical studies to correlate tumor sodium concentration with disease severity and progression are needed to evaluate the clinical applicability of ^23^Na-MRI.

## 5. Future Directions

The dietary salt (NaCl) recommendation is 3.75 g per day, to meet the 1.5 g of sodium requirement for the body’s biochemical needs. However, the average human consumption of salt has been shown to be 9 to 12 g per day. A high-salt diet is considered to be an independent risk factor for strokes and cardiovascular and renal disease, beyond its secondary impact on blood pressure [88,89,90,91]. However, certain organ systems in our body, such as skin and lymph nodes, have a natural capacity to accumulate salt, which is considered to perform a protective role in infections and activate innate and adaptive immune responses. The selective accumulation of sodium in solid organ tumors is an area of emerging interest, and not much is known about its pathophysiological importance. Inflammatory sodium channels such as ENaC and NaV1.5, due to their ability to accumulate intracellular sodium, are considered to play a role in cancer proliferation and metastasis. A correlation between hypertension and cancer needs to be more thoroughly established. Along these lines, it would be important to study the correlation between the propensity of salt concentration to skew towards pro-tumor (cancer proliferation) versus anti-tumor (immune-editing) responses. This research would allow tumor salt load to be used as a diagnostic and/or a prognostic marker. Further, salt performs an anti-tumor role in the initial phase through activation of the immune system leading to tumor elimination, followed by an antagonistic pro-tumor role in later phases through immune exhaustion. Future studies to determine the role of salt in the aggressiveness of tumors need to be performed. The clinical applicability of ^23^Na-MRI as a prognostic and/or diagnostic marker in solid organ tumors needs to be established. While the ENaC inhibitor, amiloride, cannot be directly used as an anti-cancer agent, its combinatorial role with other chemotherapeutic agents and immune checkpoint inhibitors should be determined.

## Data Availability

Not applicable.
